# Prescription drug monitoring program perceptions before and after an interprofessional workshop: a theory-informed longitudinal survey study

**DOI:** 10.3389/fdgth.2026.1746715

**Published:** 2026-02-18

**Authors:** Anne Taylor, Haley Phillippe, Brent Fox, Karen Marlowe, Renee Delaney, Garrett Aikens, Nicholas McCormick, Lindsey Hohmann

**Affiliations:** 1 Auburn University Harrison College of Pharmacy, Auburn, AL, United States; 2 Tuscaloosa Veterans Affairs (VA) Medical Center, Tuscaloosa, AL, United States

**Keywords:** healthcare provider (HCP), interprofessional, law enforcement, prescription drug monitoring program (PDMP), substance misuse, survey methods and measurement

## Abstract

**Introduction:**

The Prescription Drug Monitoring Program (PDMP) is a digital tool that can mitigate controlled substance misuse in the United States; however, it remains underutilized and end-users lack sufficient training. Thus, the purpose of this study was to assess changes in factors that influence PDMP utilization before and after an interprofessional educational workshop.

**Materials and methods:**

Fifteen two-hour interprofessional workshops were conducted from July 2022 to April 2025. Healthcare providers and law enforcement personnel in Alabama were recruited to participate via email, and data were collected at pre- and post-workshop via an anonymous online survey informed by the Unified Theory of Acceptance and Use of Technology (UTAUT). Measures included: 1) perceived usefulness; 2) ease of use; 3) social influence; 4) resources; 5) concerns; and 6) intentions regarding PDMP utilization. Differences in mean UTAUT scale scores from pre- to post-workshop were analyzed using Wilcoxon signed-rank tests, and predictors of PDMP utilization intention were analyzed using generalized estimating equations (GEE) with normal distribution and identify link function.

**Results:**

Overall (*N* = 199), mean perceived usefulness, ease of use, social factors, resources, and intentions to use the PDMP all increased (*p* < 0.001) from pre- to post-workshop, while concerns decreased (*p* = 0.007). Perceived availability of resources (*β*=0.165, 95%CI = 0.023, 0.307; *p* = 0.023) positively predicted and concerns (*β* = −0.137, 95%CI = −0.223, −0.051; *p* = 0.002) negatively predicted PDMP utilization intentions.

**Conclusion:**

Findings supports the utility of interprofessional educational interventions to increase PDMP engagement. Future studies may promote resources and alleviate concerns as key leverage points to enhance PMDP utilization.

## Introduction

Controlled substance prescription misuse is a problem nationally and in Alabama. In 2024, 13.8 million people reported misusing a psychotherapeutic drug in the past year (1). This misuse of psychotherapeutic drugs included prescription stimulants (3.9 million), prescription sedatives and tranquilizers such as benzodiazepines (4.6 million), and prescription analgesics (8 million) (1). Of individuals who misused prescription analgesics, 7.6 million misused prescription opioids, most frequently products containing hydrocodone (45.2%), oxycodone (28.6%), and codeine (26.4%) (1). Furthermore, approximately 40% of individuals who misused prescription analgesics reported obtaining them through a prescription from a single physician (1). This is especially concerning in the state of Alabama, which had an opioid prescribing rate (71.4 per 100 persons) approximately double that of the national average (37.5 per 100 persons) in 2023 (2). Thus, strategies to prevent misuse at the national and state levels are critical.

The Prescription Drug Monitoring Program (PDMP) is a tool that can mitigate controlled substance prescription misuse. The PDMP is an online database that records the prescribing and dispensing of controlled substances at the provider, pharmacy, and patient level within and across state lines (3, 4). In Alabama, the PDMP is maintained by the Alabama Department of Public Health (ADPH) and accessible by healthcare providers (prescribers, pharmacists) to assist with treatment decisions, and by law enforcement personnel to provide insights into drug overdose and diversion investigations (5). Previous research has demonstrated improved controlled substance prescribing (e.g., lower quantity and dose) (6), declines in admissions for substance use treatment (7), decreased opioid overdose mortality (8), and decreased drug-related crimes (9) as a result of PDMP adoption by states (10). Indeed, 40% of providers intended to change their opioid prescribing (11) after receiving feedback via the PDMP regarding the alignment of their prescribing with the Centers for Disease Control and Prevention (CDC) opioid prescribing guidelines (11–13).

Despite these successes, contradictory findings in some states (8), including lack of change in prescriber behavior (13) or overdose mortality (14) after PDMP implementation, underscore the need for research in this field to better understand underlying influential factors and leverage points. However, limited research investigating PDMP usability among an interprofessional audience of healthcare providers and law enforcement personnel in Alabama has been conducted to-date (11, 12). Furthermore, low rates of PDMP utilization (13) and lack of stakeholder knowledge in using the PDMP (15, 16) make end-user education and training a critical priority. Thus, the purpose of this study was to better understand current and past utilization of the PDMP, factors that influence PDMP utilization, and changes in perceived influential factors before and after an educational workshop amongst Alabama healthcare professionals and law enforcement personnel.

## Materials and methods

### Study design

A series of 15 two-hour interprofessional workshops were conducted from July 2022 to April 2025 to educate Alabama healthcare providers and law enforcement about the PDMP and provide hands-on training on how to use the PDMP. Workshops were conducted by the Auburn University Harrison College of Pharmacy (HCOP) in partnership with the ADPH. This study utilized a quasi-experimental one-group pre-post design to assess changes in participant perceptions before and after the workshops. All study procedures were reviewed and approved by the Institutional Review Board (IRB) at the primary author's institution (Protocol # 22-315 EX 2207).

### Participant recruitment and setting

Healthcare professionals (physicians, physician assistants, nurses, nurse practitioners, dentists, dental hygienists, pharmacists, pharmacy technicians) and law enforcement personnel ≥ 18 years-of-age and employed in Alabama were eligible to participate. Individuals were purposively recruited to participate in one of the 15 workshops via email distributed by the HCOP Center for Opioid Research, Education, and Outreach (COACH) and ADPH to their existing healthcare provider and law enforcement listservs. To optimize the feasibility of participant interaction with the instructor during hands-on portions of the workshop, a maximum of 20–25 participants per each of the 15 workshops was targeted. To maximize statewide participation, workshops were hosted on the HCOP campus as well as multiple community venues located throughout the state. Prescriber and pharmacist attendees received 2 h of continuing professional education credit for attending the workshop.

### Sample size estimation

A power calculation was conducted using G*Power software version 3.1.9.7 (Heinrich-Heine-Universität Düsseldorf, Düsseldorf, Germany) (17, 18). Assuming a small effect size (dz = 0.25) (19), alpha of 0.05, and power of 0.80, a minimum sample size of 134 was determined to be sufficient to assess differences in average primary outcome measures from pre- to post-workshop.

### Workshop components

A 2 h in-person workshop was developed by a content expert (GA) recruited from a clinical pharmacy practice site within the state, in consultation with HCOP faculty. This workshop was offered in-person on 15 occasions across the state of Alabama from July 2022 to April 2025, with content updates as needed to reflect the most current PDMP regulations and database. Workshop components consisted of: 1) a didactic tutorial (0.5 h); and 2) a hands-on interactive demonstration (1.5 h).

#### Didactic tutorial

Didactic programming was delivered by the content expert to familiarize participants with the basic elements of the PDMP. Didactic programming consisted of 4 topics: laws regarding PDMP reporting and querying frequencies; account maintenance; correcting common prescription errors in the PDMP; and an overview of the PDMP dashboard.

#### Hands-on interactive demonstration

During the hands-on phase of the workshop, the content expert demonstrated how to access and interpret various elements of the PDMP in real-time. Investigators worked with ADPH to create “test” patients for use during these live demonstrations. Attendees brought their own laptop computers or digital devices to log in to the PDMP and follow along as the instructor shared their screen. Key concepts demonstrated included: understanding prescriber reports including “My Rx Report” and “Prescriber Report” features; conducting queries for individual and multiple patients; and interpreting patient reports. In order to cover key concepts, interactive demonstrations were divided into 6 PDMP query activities and 6 patient case activities, with 4 discussion breaks to discuss challenges and answer questions from individual attendees.

PDMP query activities included exploring the “My Rx Report” features, “Prescriber Report” features, conducting an individual patient search, conducting a multi-state query for a specific patient, conducting a bulk patient search, and exploring the patient report features. The “My Rx Report” displays all prescriptions dispensed under a provider's DEA number, while the “Prescriber Report” displays average controlled substance prescribing [prescriptions per patient, daily morphine milligram equivalents (MME) per patient, quantity per patient, duration per patient] and number of at-risk patients compared to peers in the same specialty (5). Elements of patient reports that were explored included Narx Scores (indicating past exposure to narcotics, sedatives, and stimulants), Overdose Risk Scores (indicting likelihood of an unintentional overdose death), Rx Graphs displaying MME and lorazepam milligram equivalents (LME) prescribed over time, Prescription Summary data, and individual prescription data. Narx Scores and Overdose Risk Scores are calculated using proprietary algorithms via the NarxCare® platform (Bamboo Health, Louisville, KY, USA) (20).

Patient case activities posed various clinical scenarios utilizing test patients. Activities included assessing MME, assessing an acute pain management scenario, evaluating independent risk indicators, and exploring a patient with high-risk prescription combinations (e.g., opioids plus benzodiazepines). Factors involved in a community pharmacist PDMP query and issues specific to a dentist PDMP query were also explored.

### Data collection and measures

Data were collected via an anonymous online survey at pre-workshop (baseline) and post-workshop. Surveys were distributed via QR codes at the beginning and end of each workshop, with time built into the workshops to allow for survey completion. To maintain anonymity, a unique random code was assigned to each participant on the baseline survey via a random number generator within the Qualtrics® survey platform (Qualtrics, Provo, Utah, USA). Participants entered their assigned code on the post-survey in order to match pre- and post-workshop survey responses. The survey instruments were developed by the investigators and informed by the Unified Theory of Acceptance and Use of Technology (UTAUT) (21). Survey instruments were pre-tested among topic matter experts at the investigators’ institution (*n* = 4), and items were modified based on feedback prior to distribution. The pre- and post-survey instruments consisted of 6 primary outcome measures based on UTAUT constructs: 1) perceived usefulness of the PDMP [Performance Expectancy (PE); 3-items]; 2) ease of using the PDMP [Effort Expectancy (EE); 4-items]; 3) social factors influencing PDMP utilization [Social Influence (SI); 4-items]; 4) resources affecting PDMP utilization [Facilitating Conditions (FC); 4-items]; 5) concerns regarding PDMP utilization [Anxiety (Anx); 3-items]; and 6) intentions to utilize the PDMP [Behavioral Intentions (BI); 3-items]. UTAUT constructs were measured using 7-point Likert-type scales (1 = strongly disagree to 7 = strongly agree), with items adapted from validated measures published by Venkatesh and colleagues (21), thus ensuring comparability across prior studies. Specifically, UTAUT items were adapted by altering the wording to include the technology of interest in the current study (PDMP).

Secondarily, the baseline survey also assessed current and past utilization of the PDMP (7-items). Baseline PDMP utilization characteristics included: 1) frequency of PDMP use (3-items); 2) PDMP usage mandates by employers or licensing boards (2-items); and 3) PDMP integration into electronic medical or dispensing systems (2-items). PDMP use was measured using a combination of dichotomous multiple-choice and rating scale questions to assess having ever used the PDMP (Yes/No), using the PDMP in the past three months (Yes/No), and frequency of use in the past three months (1 = never to 5 = always). PDMP mandates and software integration were assessed through dichotomous (Yes/No) multiple-choice questions. The full survey instruments are available in Supplementary File S1.

### Data analysis

Participant demographics and outcome measures were characterized using descriptive statistics (frequencies, percentages, means, standard deviations). UTAUT scale items were summed and averaged to create total mean scale scores. Prior to calculating overall scale means, scale items were reverse coded as necessary such that higher values indicated more of a construct (e.g., more concerns, higher intentions, greater social support, higher usefulness). Missing data due to item non-response was dropped from analysis. Internal consistency of UTAUT survey scales was assessed via the Cronbach's alpha statistic. Differences in mean UTAUT scale scores from pre- to post-workshop were analyzed using two-sided Wilcoxon signed-rank tests (data were non-parametric as indicated by Komogorov-Smirnov *p* < 0.05), with effect size estimates (*r* = Z/√N) calculated and interpreted (0.1 = small, 0.3 = medium, 0.5 = large effect) based on Pautz and colleagues’ recommendations (22). Additionally, predictors [mean UTAUT scale scores and time (pre, post)] of mean PDMP utilization intention (dependent variable) were assessed via generalized estimating equations (GEE) with normal distribution, identity link function, and AR(1) working correlation matrix structure (Model 1), controlling for covariates of profession, age, and PDMP utilization frequency (Model 2). Average Likert scale scores were treated as continuous variables according to Harpe's recommendations (23), while the correlation structure utilized for the GEE was selected based on goodness-of-fit statistics [lower Quasi Likelihood under Independence Model Criterion (QIC) values]. Analyses were conducted using SPSS Statistics software version 30 (IBM Corp, Armonk, New York, USA) with *α* = 0.05.

## Results

### Participant characteristics and PDMP utilization history

Of 346 workshop attendees, 289 pre-surveys (response rate = 83.53%) and 235 post-surveys (response rate = 67.92%) were submitted. Among these, 199 individuals completed both pre- and post-surveys (useable response rate = 57.51%) (Table 1). Participants represented a range of professions, including physicians (*n* = 75, 37.7%), pharmacists (*n* = 68, 34.2%), nurses (*n* = 22, 11.1%), and dentists (*n* = 22, 11.1%). Less frequent participants included physician assistants (*n* = 2, 1.0%), pharmacy technicians (*n* = 2, 1.0%), and law enforcement personnel (*n* = 1, 0.5%).

**Table 1:** Participant characteristics and PDMP utilization history (*N* = 199)^a^.

Items	*n* (%)
Profession	
Dentist	22 (11.1)
Nurse (RN, LPN)	3 (1.5)
Nurse practitioner	22 (11.1)
Pharmacist	68 (34.2)
Pharmacy technician	2 (1.0)
Physician	75 (37.7)
Physician assistant (PA)	2 (1.0)
Law enforcement	1 (0.5)
Other	4 (2.0)
Is the PDMP integrated into your electronic medical record (EMR) software?	
Yes	53 (26.6)
No	61 (30.7)
Unsure	10 (5.0)
Is the PDMP integrated into your pharmacy dispensing software?	
Yes	43 (21.6)
No	19 (9.5)
Unsure	8 (4.0)
Have you ever utilized a PDMP database?	
Yes	182 (91.5)
No	17 (8.5)
Have you utilized a PDMP database in the last 3 months?	
Yes	154 (77.4)
No	45 (22.6)
In the past 3 months, how frequently or infrequently did you utilize the PDMP when you encountered an individual with/requesting a controlled substance?	
Never	2 (1.0)
Rarely	10 (5.0)
Sometimes	29 (14.6)
Often	56 (28.1)
Always	56 (28.1)
Does your EMPLOYER mandate that you check the PDMP in certain situations?	
Yes	68 (34.2)
No	93 (46.7)
Not applicable	37 (18.6)
Does your professional licensing board (e.g., Board of Medical Examiners, Board of Pharmacy) mandate that you check the PDMP in certain situations?	
Yes	88 (44.2)
No	84 (42.2)
Not applicable	26 (13.1)
	Mean (SD)
Age, years	54.28 (14.28)

^a^ Demographic and professional characteristics of participants (*N* = 199), along with self-reported history of Prescription Drug Monitoring Program (PDMP) use, system integration, and mandate requirements by employers and licensing boards.

The majority of participants (91.5%) reported having previously utilized a PDMP database, and 77.4% indicated PDMP use within the last three months. Among those who had used the PDMP in the past three months, 56 (28.1%) reported “often” and 56 (28.1%) reported “always” checking the PDMP when encountering a patient with/requesting a controlled substance.

Employer and licensing board mandates to check the PDMP varied. One-third (34.2%) reported employer mandates, while nearly half (44.2%) indicated that their licensing boards required PDMP checks under certain circumstances. Employer mandates were most frequently reported by pharmacists (54.4%) and nurse practitioners (45.5%), compared to 17.6% of physicians. In contrast, licensing board mandates were most often reported by nurse practitioners (72.7%) and physicians (49.3%), and less commonly by pharmacists (33.8%). Integration of the PDMP into health IT systems was inconsistent: 26.6% reported PDMP integration with their electronic medical record software, and 21.6% reported integration with pharmacy dispensing software.

### Participant beliefs and intentions regarding PDMP utilization

Baseline and post-workshop item-level survey responses demonstrated generally positive perceptions of the PDMP across the six UTAUT constructs (Table 2). Most participants agreed or strongly agreed at baseline that the PDMP was useful in their professional roles (85.5%), and this increased to 90.5% post-workshop. Similar gains were observed across measures of ease of use, social support, and availability of resources. For example, the proportion of participants agreeing or strongly agreeing that “my interaction with the PDMP is clear and understandable” increased from 58.5% pre-workshop to 78.1% post-workshop.

**Table 2:** Participant beliefs and intentions regarding PDMP utilization pre- and post-workshop (*N* = 199)^a^.

Items	*n* (%)							
	Time	1	2	3	4	5	6	7
Usefulness								
I find the PDMP useful in my job.	Pre	6 (3.1)	3 (1.5)	1 (0.5)	17 (8.7)	21 (10.7)	70 (35.7)	78 (39.8)
	Post	1 (0.5)	2 (1.0)	2 (1.0)	14 (7.1)	17 (8.6)	71 (35.9)	91 (46.0)
Using the PDMP enables me to accomplish tasks more quickly.	Pre	11 (5.5)	14 (7.0)	18 (9.0)	44 (22.1)	28 (14.1)	46 (23.1)	38 (19.1)
	Post	3 (1.5)	11 (5.6)	11 (5.6)	31 (15.7)	28 (14.2)	62 (31.5)	51 (25.9)
Using the PDMP increases my productivity.	Pre	9 (4.6)	18 (9.2)	18 (9.2)	56 (28.7)	39 (20.0)	27 (13.8)	28 (14.4)
	Post	4 (2.0)	13 (6.6)	10 (5.1)	40 (20.3)	32 (16.2)	51 (25.9)	47 (23.9)
Ease of use								
My interaction with the PDMP is clear and understandable.	Pre	4 (2.1)	4 (2.1)	9 (4.6)	27 (13.8)	37 (19.0)	71 (36.4)	43 (22.1)
	Post	1 (0.5)	2 (1.0)	2 (1.0)	13 (6.6)	25 (12.7)	82 (41.6)	72 (36.5)
It is easy for me to become skillful at using the PDMP.	Pre	3 (1.5)	4 (2.1)	8 (4.1)	39 (20.1)	35 (18.0)	61 (31.4)	44 (22.7)
	Post	1 (0.5)	1 (0.5)	2 (1.0)	11 (5.6)	29 (14.7)	81 (41.1)	72 (36.5)
I find the PDMP easy to use.	Pre	1 (0.5)	4 (2.1)	9 (4.8)	27 (14.4)	42 (22.3)	65 (34.6)	40 (21.3)
	Post	1 (0.5)	1 (0.5)	1 (0.5)	14 (7.2)	34 (17.5)	76 (39.2)	67 (34.5)
Learning to operate the PDMP is easy for me.	Pre	3 (1.5)	9 (4.6)	31 (16.0)	0 (0)	41 (20.6)	72 (37.1)	38 (19.6)
	Post	1 (0.5)	6 (3.0)	0 (0)	12 (6.1)	31 (15.7)	84 (42.6)	63 (32.0)
Social factors								
People who influence my behavior think that I should use the PDMP.	Pre	2 (1.0)	7 (3.6)	6 (3.1)	68 (35.1)	19 (9.8)	58 (29.9)	34 (17.5)
	Post	0 (0)	5 (2.6)	2 (1.0)	54 (27.7)	24 (12.3)	60 (30.8)	50 (25.6)
People who are important to me think that I should use the PDMP.	Pre	3 (1.6)	8 (4.1)	4 (2.1)	67 (34.7)	26 (13.5)	59 (30.6)	26 (13.5)
	Post	0 (0)	4 (2.0)	3 (1.5)	55 (27.8)	21 (10.6)	66 (33.3)	49 (24.7)
My professional colleagues have been helpful in the use of the PDMP.	Pre	2 (1.0)	7 (3.6)	9 (4.6)	72 (36.9)	26 (13.3)	48 (24.6)	31 (15.9)
	Post	0 (0)	7 (3.6)	10 (5.1)	50 (25.6)	28 (14.4)	57 (29.2)	43 (22.1)
In general, my workplace has supported the use of the PDMP.	Pre	1 (0.5)	2 (1.0)	3 (1.5)	33 (16.9)	27 (13.8)	64 (32.8)	65 (33.3)
	Post	0 (0)	2 (1.0)	7 (3.6)	27 (13.7)	18 (9.1)	71 (36.0)	72 (36.5)
Resources								
I have the resources necessary to use the PDMP.	Pre	1 (0.5)	2 (1.0)	3 (1.5)	22 (11.3)	21 (10.8)	80 (41.0)	66 (33.8)
	Post	0 (0)	1 (0.5)	0 (0)	9 (4.6)	21 (10.7)	82 (41.8)	83 (42.3)
I have the knowledge necessary to use the PDMP.	Pre	1 (0.5)	2 (1.0)	7 (3.6)	28 (14.4)	26 (13.3)	72 (36.9)	59 (30.3)
	Post	0 (0)	0 (0)	3 (1.5)	8 (4.1)	22 (11.2)	82 (41.6)	82 (41.6)
The PDMP is not compatible with other systems I use^b^.	Pre	24 (12.2)	62 (31.5)	15 (7.6)	59 (29.9)	15 (7.6)	17 (8.6)	5 (2.5)
	Post	35 (17.6)	50 (25.4)	15 (7.6)	56 (28.4)	8 (4.1)	26 (13.2)	7 (3.6)
A specific person (or group) is available for assistance with PDMP difficulties.	Pre	9 (4.6)	28 (14.4)	14 (7.2)	86 (44.3)	15 (7.7)	29 (14.9)	13 (6.7)
	Post	5 (2.6)	19 (9.7)	7 (3.6)	50 (25.5)	12 (6.1)	65 (33.2)	38 (19.4)
Concerns								
I feel apprehensive about using the PDMP.	Pre	45 (23.1)	76 (39.0)	16 (8.2)	30 (15.4)	15 (7.7)	10 (5.1)	3 (1.5)
	Post	65 (33.2)	71 (36.2)	15 (7.7)	19 (9.7)	10 (5.1)	13 (6.6)	3 (1.5)
I hesitate to use the PDMP for fear of making mistakes I cannot correct.	Pre	63 (32.5)	71 (36.6)	20 (10.3)	25 (12.9)	9 (4.6)	4 (2.1)	2 (1.0)
	Post	73 (37.2)	74 (37.8)	10 (5.1)	16 (8.2)	11 (5.6)	9 (4.6)	3 (1.5)
The PDMP is somewhat intimidating to me.	Pre	51 (26.3)	77 (39.7)	12 (6.2)	24 (12.4)	18 (9.3)	11 (5.7)	1 (0.5)
	Post	66 (33.7)	72 (36.7)	11 (5.6)	20 (10.2)	12 (6.1)	11 (5.6)	4 (2.0)
Intentions								
I intend to use the PDMP in the next 3 months.	Pre	2 (1.0)	4 (2.1)	2 (1.0)	29 (15.1)	13 (6.8)	57 (29.7)	85 (44.3)
	Post	1 (0.5)	2 (1.0)	2 (1.0)	16 (8.3)	12 (6.2)	67 (34.7)	93 (48.2)
I predict I will use the PDMP in the next 3 months.	Pre	2 (1.0)	5 (2.6)	1 (0.5)	25 (13.0)	12 (6.2)	61 (31.6)	87 (45.1)
	Post	1 (0.5)	1 (0.5)	2 (1.0)	16 (8.2)	14 (7.1)	67 (34.2)	95 (48.5)
I plan to use the PDMP in the next 3 months.	Pre	2 (1.0)	5 (2.6)	0 (0)	32 (16.7)	12 (6.3)	53 (27.6)	88 (45.8)
	Post	1 (0.5)	1 (0.5)	2 (1.0)	16 (8.3)	18 (9.3)	62 (32.1)	93 (48.2)

^a^ Pre- and post-workshop responses to survey items assessing UTAUT constructs (usefulness, ease of use, social influence, facilitating conditions/resources, concerns, and behavioral intentions). Items were rated on a 7-point Likert scale. Values represent counts and percentages of participants selecting each response option. 1 = Strongly Disagree; 2 = Disagree; 3 = Somewhat Disagree; 4 = Neutral; 5 = Somewhat Agree; 6 = Agree; 7 = Strongly Agree.

^b^ Reverse-coded item.

Concerns about PDMP use decreased following the workshops. At baseline, nearly half of participants (49.8%) expressed some degree of apprehension, hesitation, or intimidation about PDMP use, compared to 36.0% post-workshop.

Intentions to use the PDMP were high at baseline and remained high post-workshop. Prior to the workshop, 81.8% of participants agreed or strongly agreed that they intended to use the PDMP in the next three months; this proportion increased to 87.6% after the workshop.

### Changes in UTAUT constructs pre- and post-workshop

As shown in Table 3, overall mean scale scores improved significantly across all six UTAUT constructs following the workshops. Perceived usefulness increased from a mean of 5.05 (SD = 1.38) pre-workshop to 5.54 (SD = 1.21) post-workshop (*p* < 0.001, effect size = 0.48). Ease of use increased from 5.42 (SD = 1.24) to 5.96 (SD = 1.03) (*p* < 0.001, effect size = 0.55). Social factors, resources, and intentions to use the PDMP all demonstrated significant improvements (*p* < 0.001), while concerns decreased significantly from 2.53 (SD = 1.37) to 2.39 (SD = 1.50) (*p* = 0.007, effect size = 0.19). Internal consistency of survey scales was acceptable with Cronbach's alpha ranging from 0.801–0.979 across pre and post time-points, with the exception of low internal consistency for the resources scale (0.364–0.491), indicating a measurement limitation within this construct.

**Table 3:** Changes in UTAUT factors pre- and post-workshop (*N* = 199)^a^.

Measures	Cronbach's alpha		Mean (SD) Median (IQR)		Effect Size	*p*-value
	Pre	Post	Pre	Post		
Usefulness	0.828	0.801	5.05 (1.375) 5.00 (4.33, 6.00)	5.54 (1.205) 5.67 (4.67, 6.42)	0.481	<0.001
Ease of use	0.956	0.967	5.42 (1.244) 5.75 (4.50, 6.00)	5.96 (1.032) 6.00 (5.50, 7.00)	0.554	<0.001
Social factors	0.862	0.876	5.19 (1.142) 5.25 (4.38, 6.00)	5.50 (1.098) 5.75 (4.75, 6.25)	0.413	<0.001
Resources	0.364	0.491	5.10 (0.845) 5.00 (4.50, 5.75)	5.52 (0.860) 5.50 (5.00, 6.25)	0.487	<0.001
Concerns	0.891	0.945	2.53 (1.368) 2.00 (1.33, 3.67)	2.39 (1.504) 2.00 (1.00, 3.00)	0.192	0.007
Intentions	0.979	0.970	5.91 (1.329) 6.00 (5.33,7.00)	6.15 (1.066) 6.00 (6.00, 7.00)	0.308	<0.001

^a^ Comparison of pre- and post-workshop mean scores, standard deviations (SD), medians, interquartile ranges (IQR), effect sizes (*r*), and *p*-values for UTAUT constructs (usefulness, ease of use, social influence, facilitating conditions/resources, concerns, and intentions). Internal consistency (Cronbach's alpha) is reported for each construct.

### Predictors of PDMP utilization intention

In adjusted GEE analysis (Table 4, Model 2), perceived availability of resources (*β* = 0.165, 95%CI = 0.023, 0.307; *p* = 0.023) and PDMP utilization frequency in the past 3 months (*β* = 0.196, 95%CI = 0.070, 0.322; *p* = 0.002) were positive predictors of future intention to utilize the PDMP. In contrast, concerns (*β* = −0.137, 95%CI = −0.223, −0.051; *p* = 0.002) negatively predicted PDMP utilization intentions. Controlling for covariates in the adjusted model (Model 2) did not alter statistical significance of predictors compared to the unadjusted model (Model 1). These associations should be interpreted in the context of self-reported perceptions and intentions rather than observed behavior.

**Table 4:** Predictors of PDMP utilization intention (*N* = 199)^a^.

Model 1: QIC = 142.691, QICC = 132.874			
Measures	*β*	95%CI	*p*-value
Usefulness	0.085	−0.010, 0.179	0.080
Ease of use	0.084	−0.085, 0.253	0.330
Social influence	0.090	−0.012, 0.192	0.084
Resources	0.167	0.017, 0.317	**0**.030*
Concerns	−0.165	−0.257, −0.073	**<0**.001*
Time	−0.103	−0.215, 0.010	0.075
Model 2: QIC = 147.252, QICC = 130.758			
Usefulness	0.060	−0.028, 0.149	0.183
Ease of use	0.082	−0.071, 0.235	0.293
Social influence	0.026	−0.071, 0.123	0.602
Resources	0.165	0.023, 0.307	**0**.023*
Concerns	−0.137	−0.223, −0.051	**0**.002*
Time	−0.063	−0.178, 0.051	0.278
Profession	−0.033	−0.093, 0.027	0.276
Age	−0.005	−0.013, 0.003	0.249
PDMP utilization frequency	0.196	0.070, 0.322	**0**.002*

^a^ Analysis of predictors of PDMP utilization intention via generalized estimating equations (GEE) with normal distribution, identity link function, and AR(1) working correlation matrix structure. Working correlation structure and model parameters were selected based on QIC (Quasi Likelihood under Independence Model Criterion) and QICC (Corrected Quasi Likelihood under Independence Model Criterion) goodness of fit statistics, respectively. Model 1: Dependent variable = mean intention scale score. Independent variables = mean usefulness, ease of use, social influence, availability of resources, and concerns scale scores; time [pre- [1] and post- [2] workshop]. Model 2: Dependent variable = mean intention scale score. Independent variables = mean usefulness, ease of use, social influence, availability of resources, and concerns scale scores; time [pre- [1] and post- [2] workshop]. Controlling for covariates of profession, age, and PDMP utilization frequency over the past 3 months (1 = never, 5 = always).

* The bold asterisk indicates statistical significance at the 0.05 level.

## Discussion

This study evaluated the impact of interprofessional townhall-style workshops on healthcare professionals’ and law enforcement personnel's perceptions and intentions regarding PDMP use in Alabama. Findings demonstrate that participation was associated with significant improvements across all six UTAUT constructs, including perceived usefulness, ease of use, social influences, available resources, and behavioral intentions, while concerns decreased. These results suggest that brief, interactive and practice-focused educational sessions can meaningfully enhance stakeholders’ confidence and willingness to engage with the PDMP.

Prior to the workshops, the majority of participants reported prior PDMP use, yet many expressed apprehension and highlighted barriers such as a lack of integration with electronic systems. These challenges align with previously reported barriers to PDMP adoption at both the national and state levels (24–27). After the workshops, participants reported greater confidence in their potential ability to use the PDMP and stronger perceptions of organizational and social support for its future use. Notably, while baseline intentions to use the PDMP were already high, intentions increased further post-intervention, underscoring the reinforcing effect of hands-on training.

Our findings are consistent with prior studies demonstrating that PDMP education and training can improve provider knowledge, attitudes, and self-efficacy related to future PDMP use (28, 29). For example, previous evaluations of targeted PDMP training programs in clinical and community settings have similarly shown improvements in perceived ease of use and decreased concerns (30). The present study extends this work by engaging a broad array of healthcare professionals and law enforcement in a shared learning environment, which may foster cross sector collaboration in addressing controlled substance misuse.

These results highlight the value of structured, interactive PDMP workshops as a scalable strategy to address underutilization. Incorporating both didactic and hands-on components appears particularly effective for reducing apprehension and improving perceived resources. Given that PDMP integration into electronic health and pharmacy dispensing systems remained inconsistent, future policy initiatives should prioritize technological integrations alongside education. Additionally, the mixed reports of employer and licensing board mandates suggest opportunities for regulatory alignment to support consistent future PDMP use.

This study has several limitations. First, the participants self-selected into workshops, which may have overrepresented individuals who were already motivated to engage with the PDMP. Second, the survey relied on self-reporting which may not reflect actual past or intended future PDMP utilization behavior. Third, while internal consistency for most constructs was strong, reliability for the resources construct was low, suggesting further refinement of measurement tools is warranted. Additionally, the study was conducted in Alabama and may not be generalizable to other states with different PDMP structures or mandates. Finally, law enforcement participation in the workshops was low, and thus findings largely reflect healthcare professionals. In order to reach a statewide audience efficiently and within budget, the current study purposively recruited providers and law enforcement via existing email listservs; future studies should explore multiple avenues of recruitment to enhance law enforcement engagement such as social media advertisements, personal selling, direct mail marketing, and obtaining buy-in from opinion leaders across law enforcement departments in the state.

## Conclusion

Overall, participation in interprofessional workshops was associated with greater perceived usefulness, ease of use, social support, and resource availability for PDMP utilization, as well as reduced apprehension and stronger behavioral intentions to use the PDMP among healthcare professionals and law enforcement personnel in Alabama. These improvements in perceived readiness and intentions support the utility of targeted educational interventions to increase PDMP engagement, although future studies should assess actual PDMP utilization outcomes. Continued efforts to expand training opportunities, enhance system integration and align organizational and regulatory mandates are critical to optimizing PDMP utilization as a strategy to mitigate controlled substance misuse.

## Data Availability

The datasets presented in this article are not readily available because Institutional Review Board (IRB) restrictions apply. Requests to access the datasets should be directed to Lindsey Hohmann, lah0036@auburn.edu.
