# Supplementary material for: Prescription drug monitoring program perceptions before and after an interprofessional workshop: a theory-informed longitudinal survey study
Source: Front Digit Health. 2026 Feb 18;8:1746715. doi: 10.3389/fdgth.2026.1746715 (PMC12958023; doi:10.3389/fdgth.2026.1746715)
Supplement: Supplementary file 1 [file Supplementaryfile1.docx]

**PRESCRIPTION DRUG MONITORING PROGRAM (PDMP) WORKSHOP**

***Pre-Survey***

1. **SURVEY CODE**
2. In order for your responses to remain anonymous, the following code will serve as your unique survey identifier.

**Please write down, copy, take a picture or screenshot of the survey code below. Save this code to enter on the post-program survey.**

**YOUR UNIQUE SURVEY CODE:** _____[Embed random 4-digit code in Qualtrics]_________

1. **INFORMATION ABOUT YOU**
2. Please indicate your profession:

🞏 Dentist 🞏 Dental hygienist

🞏 Nurse (RN, LPN) 🞏 Nurse practitioner

🞏 Pharmacist 🞏 Pharmacy technician

🞏 Physician 🞏 Physician assistant (PA)

🞏 Law enforcement 🞏 Other. Please specify: __________________

1. Please enter your age in years: _________________
2. **CURRENT AND PAST UTILIZATION OF THE PDMP**
3. ***[Display if Q2 “Dentist,” “Dental hygienist,” “Nurse,” “Nurse practitioner,” “Physician,” or “PA” is selected]*** Is the PDMP integrated into your electronic medical record (EMR) software?

🞏 Yes 🞏 No 🞏 Unsure

1. ***[Display if Q2 “Pharmacist” or “Pharmacy technician” is selected]*** Is the PDMP integrated into your pharmacy dispensing software?

🞏 Yes 🞏 No 🞏 Unsure

1. Have you ever utilized a PDMP database?

🞏 Yes 🞏 No

1. Have you utilized a PDMP database in the last 3 months?

🞏 Yes 🞏 No

1. ***[Do not Display if Q7 “No” is selected]*** In the past 3 months, how frequently or infrequently did you utilize the PDMP when you encountered an individual with/requesting a controlled substance?

🞏 Never 🞏 Rarely 🞏 Sometimes 🞏 Often 🞏 Always 🞏 Not applicable

1. Does your EMPLOYER mandate that you check the PDMP in certain situations?

🞏 Yes. Please explain: _____________________ 🞏 No 🞏 Not applicable

1. ***[Do not Display if Q2 “Law Enforcement” is selected]*** Does your professional licensing board (e.g., Board of Medical Examiners, Board of Pharmacy) mandate that you check the PDMP in certain situations?

🞏 Yes. Please explain: _____________________ 🞏 No 🞏 Not applicable

1. **FACTORS AFFECTING UTILIZATION OF THE PDMP**

One a scale of 1 to 7, please indicate your level of agreement or disagreement with the following statements regarding factors that affect / would affect your use of the PDMP in your workplace, where 1=strongly disagree and 7=strongly agree.

If you do not currently utilize the PDMP in your workplace, imagine a scenario in which the PDMP is being implemented in your workplace.

1. ***Usefulness of the PDMP in your workplace:***

|  | **Strongly Disagree (1)** | **Disagree** | **Somewhat Disagree** | **Neutral** | **Somewhat Agree** | **Agree** | **Strongly Agree (7)** |
| --- | --- | --- | --- | --- | --- | --- | --- |
| I find the PDMP useful in my job. | 🞏 | 🞏 | 🞏 | 🞏 | 🞏 | 🞏 | 🞏 |
| Using the PDMP enables me to accomplish tasks more quickly. | 🞏 | 🞏 | 🞏 | 🞏 | 🞏 | 🞏 | 🞏 |
| Using the PDMP increases my productivity. | 🞏 | 🞏 | 🞏 | 🞏 | 🞏 | 🞏 | 🞏 |

1. ***Ease of using the PDMP in your workplace:***

|  | **Strongly Disagree (1)** | **Disagree** | **Somewhat Disagree** | **Neutral** | **Somewhat Agree** | **Agree** | **Strongly Agree (7)** |
| --- | --- | --- | --- | --- | --- | --- | --- |
| My interaction with the PDMP is clear and understandable. | 🞏 | 🞏 | 🞏 | 🞏 | 🞏 | 🞏 | 🞏 |
| It is easy for me to become skillful at using the PDMP. | 🞏 | 🞏 | 🞏 | 🞏 | 🞏 | 🞏 | 🞏 |
| I find the PDMP easy to use. | 🞏 | 🞏 | 🞏 | 🞏 | 🞏 | 🞏 | 🞏 |
| Learning to operate the PDMP is easy for me. | 🞏 | 🞏 | 🞏 | 🞏 | 🞏 | 🞏 | 🞏 |

1. ***Social factors influencing PDMP utilization in your workplace:***

|  | **Strongly Disagree (1)** | **Disagree** | **Somewhat Disagree** | **Neutral** | **Somewhat Agree** | **Agree** | **Strongly Agree (7)** |
| --- | --- | --- | --- | --- | --- | --- | --- |
| People who influence my behavior think that I should use the PDMP. | 🞏 | 🞏 | 🞏 | 🞏 | 🞏 | 🞏 | 🞏 |
| People who are important to me think that I should use the PDMP. | 🞏 | 🞏 | 🞏 | 🞏 | 🞏 | 🞏 | 🞏 |
| My professional colleagues have been helpful in the use of the PDMP. | 🞏 | 🞏 | 🞏 | 🞏 | 🞏 | 🞏 | 🞏 |
| In general, my workplace has supported the use of the PDMP. | 🞏 | 🞏 | 🞏 | 🞏 | 🞏 | 🞏 | 🞏 |

1. ***Resources affecting PDMP utilization in your workplace:***

|  | **Strongly Disagree (1)** | **Disagree** | **Somewhat Disagree** | **Neutral** | **Somewhat Agree** | **Agree** | **Strongly Agree (7)** |
| --- | --- | --- | --- | --- | --- | --- | --- |
| I have the resources necessary to use the PDMP. | 🞏 | 🞏 | 🞏 | 🞏 | 🞏 | 🞏 | 🞏 |
| I have the knowledge necessary to use the PDMP. | 🞏 | 🞏 | 🞏 | 🞏 | 🞏 | 🞏 | 🞏 |
| The PDMP is not compatible with other systems I use.^r^ | 🞏 | 🞏 | 🞏 | 🞏 | 🞏 | 🞏 | 🞏 |
| A specific person (or group) is available for assistance with PDMP difficulties. | 🞏 | 🞏 | 🞏 | 🞏 | 🞏 | 🞏 | 🞏 |

^r^ Reverse-coded item.

1. ***Concerns regarding PDMP utilization in your workplace:***

|  | **Strongly Disagree (1)** | **Disagree** | **Somewhat Disagree** | **Neutral** | **Somewhat Agree** | **Agree** | **Strongly Agree (7)** |
| --- | --- | --- | --- | --- | --- | --- | --- |
| I feel apprehensive about using the PDMP. | 🞏 | 🞏 | 🞏 | 🞏 | 🞏 | 🞏 | 🞏 |
| I hesitate to use the PDMP for fear of making mistakes I cannot correct. | 🞏 | 🞏 | 🞏 | 🞏 | 🞏 | 🞏 | 🞏 |
| The PDMP is somewhat intimidating to me. | 🞏 | 🞏 | 🞏 | 🞏 | 🞏 | 🞏 | 🞏 |

1. ***Intentions to utilize the PDMP in your workplace:***

|  | **Strongly Disagree (1)** | **Disagree** | **Somewhat Disagree** | **Neutral** | **Somewhat Agree** | **Agree** | **Strongly Agree (7)** |
| --- | --- | --- | --- | --- | --- | --- | --- |
| I intend to use the PDMP in the next 3 months. | 🞏 | 🞏 | 🞏 | 🞏 | 🞏 | 🞏 | 🞏 |
| I predict I will use the PDMP in the next 3 months. | 🞏 | 🞏 | 🞏 | 🞏 | 🞏 | 🞏 | 🞏 |
| I plan to use the PDMP in the next 3 months. | 🞏 | 🞏 | 🞏 | 🞏 | 🞏 | 🞏 | 🞏 |

**PRESCRIPTION DRUG MONITORING PROGRAM (PDMP) WORKSHOP**

***Post-Survey***

1. **SURVEY CODE**
2. Please provide your unique survey code.

**YOUR UNIQUE SURVEY CODE:** ______________________________________________

1. **FACTORS AFFECTING UTILIZATION OF THE PDMP**

One a scale of 1 to 7, please indicate your level of agreement or disagreement with the following statements regarding factors that affect / would affect your use of the PDMP in your workplace, with 1 being strongly disagree and 7 being strongly agree.

If you do not currently utilize the PDMP in your workplace, imagine a scenario in which the PDMP is being implemented in your workplace.

1. ***Usefulness of the PDMP in your workplace:***

|  | **Strongly Disagree (1)** | **Disagree** | **Somewhat Disagree** | **Neutral** | **Somewhat Agree** | **Agree** | **Strongly Agree (7)** |
| --- | --- | --- | --- | --- | --- | --- | --- |
| I find the PDMP useful in my job. | 🞏 | 🞏 | 🞏 | 🞏 | 🞏 | 🞏 | 🞏 |
| Using the PDMP enables me to accomplish tasks more quickly. | 🞏 | 🞏 | 🞏 | 🞏 | 🞏 | 🞏 | 🞏 |
| Using the PDMP increases my productivity. | 🞏 | 🞏 | 🞏 | 🞏 | 🞏 | 🞏 | 🞏 |

1. ***Ease of using the PDMP in your workplace:***

|  | **Strongly Disagree (1)** | **Disagree** | **Somewhat Disagree** | **Neutral** | **Somewhat Agree** | **Agree** | **Strongly Agree (7)** |
| --- | --- | --- | --- | --- | --- | --- | --- |
| My interaction with the PDMP is clear and understandable. | 🞏 | 🞏 | 🞏 | 🞏 | 🞏 | 🞏 | 🞏 |
| It is easy for me to become skillful at using the PDMP. | 🞏 | 🞏 | 🞏 | 🞏 | 🞏 | 🞏 | 🞏 |
| I find the PDMP easy to use. | 🞏 | 🞏 | 🞏 | 🞏 | 🞏 | 🞏 | 🞏 |
| Learning to operate the PDMP is easy for me. | 🞏 | 🞏 | 🞏 | 🞏 | 🞏 | 🞏 | 🞏 |

1. ***Social factors influencing PDMP utilization in your workplace:***

|  | **Strongly Disagree (1)** | **Disagree** | **Somewhat Disagree** | **Neutral** | **Somewhat Agree** | **Agree** | **Strongly Agree (7)** |
| --- | --- | --- | --- | --- | --- | --- | --- |
| People who influence my behavior think that I should use the PDMP. | 🞏 | 🞏 | 🞏 | 🞏 | 🞏 | 🞏 | 🞏 |
| People who are important to me think that I should use the PDMP. | 🞏 | 🞏 | 🞏 | 🞏 | 🞏 | 🞏 | 🞏 |
| My professional colleagues have been helpful in the use of the PDMP. | 🞏 | 🞏 | 🞏 | 🞏 | 🞏 | 🞏 | 🞏 |
| In general, my workplace has supported the use of the PDMP. | 🞏 | 🞏 | 🞏 | 🞏 | 🞏 | 🞏 | 🞏 |

1. ***Resources affecting PDMP utilization in your workplace:***

|  | **Strongly Disagree (1)** | **Disagree** | **Somewhat Disagree** | **Neutral** | **Somewhat Agree** | **Agree** | **Strongly Agree (7)** |
| --- | --- | --- | --- | --- | --- | --- | --- |
| I have the resources necessary to use the PDMP. | 🞏 | 🞏 | 🞏 | 🞏 | 🞏 | 🞏 | 🞏 |
| I have the knowledge necessary to use the PDMP. | 🞏 | 🞏 | 🞏 | 🞏 | 🞏 | 🞏 | 🞏 |
| The PDMP is not compatible with other systems I use.^r^ | 🞏 | 🞏 | 🞏 | 🞏 | 🞏 | 🞏 | 🞏 |
| A specific person (or group) is available for assistance with PDMP difficulties. | 🞏 | 🞏 | 🞏 | 🞏 | 🞏 | 🞏 | 🞏 |

^r^ Reverse-coded item.

1. ***Concerns regarding PDMP utilization in your workplace:***

|  | **Strongly Disagree (1)** | **Disagree** | **Somewhat Disagree** | **Neutral** | **Somewhat Agree** | **Agree** | **Strongly Agree (7)** |
| --- | --- | --- | --- | --- | --- | --- | --- |
| I feel apprehensive about using the PDMP. | 🞏 | 🞏 | 🞏 | 🞏 | 🞏 | 🞏 | 🞏 |
| I hesitate to use the PDMP for fear of making mistakes I cannot correct. | 🞏 | 🞏 | 🞏 | 🞏 | 🞏 | 🞏 | 🞏 |
| The PDMP is somewhat intimidating to me. | 🞏 | 🞏 | 🞏 | 🞏 | 🞏 | 🞏 | 🞏 |

1. ***Intentions to utilize the PDMP in your workplace:***

|  | **Strongly Disagree (1)** | **Disagree** | **Somewhat Disagree** | **Neutral** | **Somewhat Agree** | **Agree** | **Strongly Agree (7)** |
| --- | --- | --- | --- | --- | --- | --- | --- |
| I intend to use the PDMP in the next 3 months. | 🞏 | 🞏 | 🞏 | 🞏 | 🞏 | 🞏 | 🞏 |
| I predict I will use the PDMP in the next 3 months. | 🞏 | 🞏 | 🞏 | 🞏 | 🞏 | 🞏 | 🞏 |
| I plan to use the PDMP in the next 3 months. | 🞏 | 🞏 | 🞏 | 🞏 | 🞏 | 🞏 | 🞏 |
